# Plasticity of the 340-Loop in Influenza Neuraminidase Offers New Insight for Antiviral Drug Development

**DOI:** 10.3390/ijms21165655

**Published:** 2020-08-06

**Authors:** Nanyu Han, Justin Tze Yang Ng, Yanpeng Li, Yuguang Mu, Zunxi Huang

**Affiliations:** 1 School of Life Sciences, Yunnan Normal University, Kunming 650500, China; ha0001yu@e.ntu.edu.sg; 2 Key Laboratory of Enzyme Engineering, Yunnan Normal University, Kunming 650500, China; 3 Engineering Research Center of Sustainable and Utilization of Biomass Energy, Ministry of Education, Kunming 650500, China; 4 School of Biological Sciences, Nanyang Technological University, Singapore 639798, Singapore; ngtz0013@e.ntu.edu.sg; 5 Zhaoqing Institute of Biotechnology Co., Ltd., Zhaoqing 526238, China; xjdwlab@163.com

**Keywords:** influenza, neuraminidase, 340-cavity, molecular dynamics simulations

## Abstract

The recently discovered 340-cavity in influenza neuraminidase (NA) N6 and N7 subtypes has introduced new possibilities for rational structure-based drug design. However, the plasticity of the 340-loop (residues 342–347) and the role of the 340-loop in NA activity and substrate binding have not been deeply exploited. Here, we investigate the mechanism of 340-cavity formation and demonstrate for the first time that seven of nine NA subtypes are able to adopt an open 340-cavity over 1.8 μs total molecular dynamics simulation time. The finding that the 340-loop plays a role in the sialic acid binding pathway suggests that the 340-cavity can function as a druggable pocket. Comparing the open and closed conformations of the 340-loop, the side chain orientation of residue 344 was found to govern the formation of the 340-cavity. Additionally, the conserved calcium ion was found to substantially influence the stability of the 340-loop. Our study provides dynamical evidence supporting the 340-cavity as a druggable hotspot at the atomic level and offers new structural insight in designing antiviral drugs.

## 1. Introduction

Since the beginning of the last century, multiple pandemics caused by influenza viruses have severely impacted public health [1]. Although vaccines and antiviral drugs have been developed to prevent and control upcoming influenza outbreaks, unpredictable novel strains and constantly mutating existing strains still pose a great threat to human life [2]. In 2020, COVID-19, caused by the SARS-CoV-2 coronavirus, has already affected more than 200 countries, taken more than 500,000 lives, and instigated a significant global economic downturn [3]. Influenza, which also spreads readily via respiratory droplets, cannot be neglected for its high morbidity and mortality [4]. New antiviral drugs are a promising and straightforward approach to treating influenza by reducing case severity and transmission, which can be particularly impactful for responding to new outbreaks [5]. Neuraminidase (NA), which releases progeny virions by cleaving the terminal sialic acid (SA) residues on the infected cell surface, is considered the most promising target for antiviral drug development [6]. Therefore, exploring the structural features of NAs and identifying the SA binding path are of great importance in designing anti-influenza drugs [7,8].

Nine NA alleles are classified into two groups according to phylogenic and structural differences (group-1: N1, N4, N5, N8; group-2: N2, N3, N6, N7, N9) [9]. The crystal structures of group-1 NAs contain a unique characteristic, the 150-cavity, which can be exploited to develop new anti-influenza drugs [10]. It is noteworthy that an additional pocket, the 340-cavity, adjacent to the active site of NA capped by the 340-loop (residues 342–347) was observed in the crystal structures of N6 and N7 [11]. Our group discovered that the hydrogen bond between G342 and Q296 and the calcium ion bound in the 340-loop contribute to the special conformation of the N7 340-cavity [12]. However, questions regarding whether the 340-cavity is prevalent in NAs, whether the 340-loop participates in the substrate binding process, and the structural dynamics of the 340-loop in other NA subtypes have never been explored.

To answer the above questions, normal molecular dynamics (MD) simulations were performed on N1 to N9 subtypes in calcium-bound and calcium-free systems. Additionally, the binding mechanism of natural substrate SA was identified by sliding binding-box docking. Our study reveals that seven of the nine NA subtypes exhibit an open 340-cavity, the calcium ion plays an important role in maintaining the conformation of the 340-loop, and the 340-loop is a canonical approach site in the SA binding mechanism. Our results indicate that the 340-loop is a critical motif in NA, and the 340-cavity is a valuable hotspot for anti-influenza drug design.

## 2. Results

### 2.1. N1, N3, N4, N5, N6, N7, and N8 Exhibit an Open 340-Cavity

**Figure 1:**
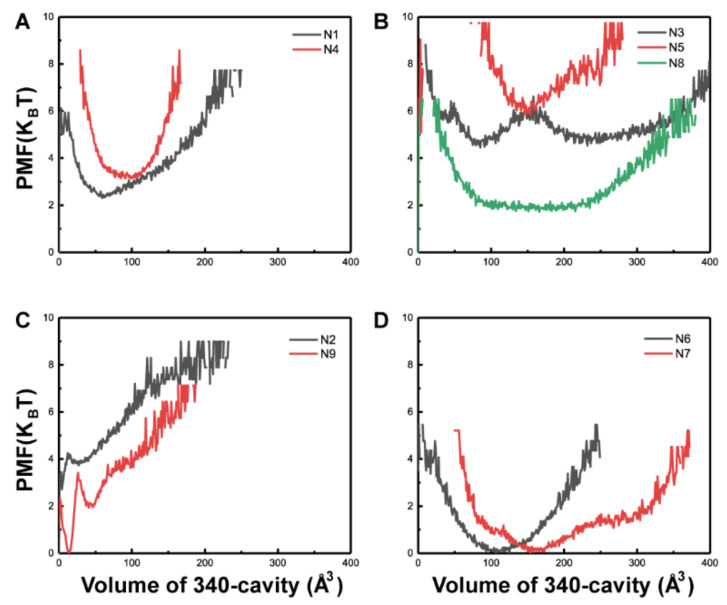
Potential of mean force (PMF) of nine neuraminidase (NA) subtypes based on 340-cavity volume calculation. Potential of mean force (PMF) based on the volume of the 340-cavity in N1 and N4 (**A**), N3, N5, and N8 (**B**), N2 and N9 (**C**), N6 and N7 (**D**).

To probe 340-cavity formation in NAs, the volumes of the 340-cavity for all NA subtypes were calculated using POVME. Maps of the potential of mean force (PMF) from N1 to N9 as a function of 340-cavity volume were constructed based on the simulation data (Figure 1). To compare the size of the 340-cavity, the volume of the N7 crystal structure 340-cavity, 102 Å^3^, was given as a reference. It revealed that most of the NA subtypes, except N2 and N9, are able to adopt open 340-cavity conformations in normal MD simulations. Moreover, the NA subtypes can be divided into three types based on their 340-cavity volume.

**Figure 2:**
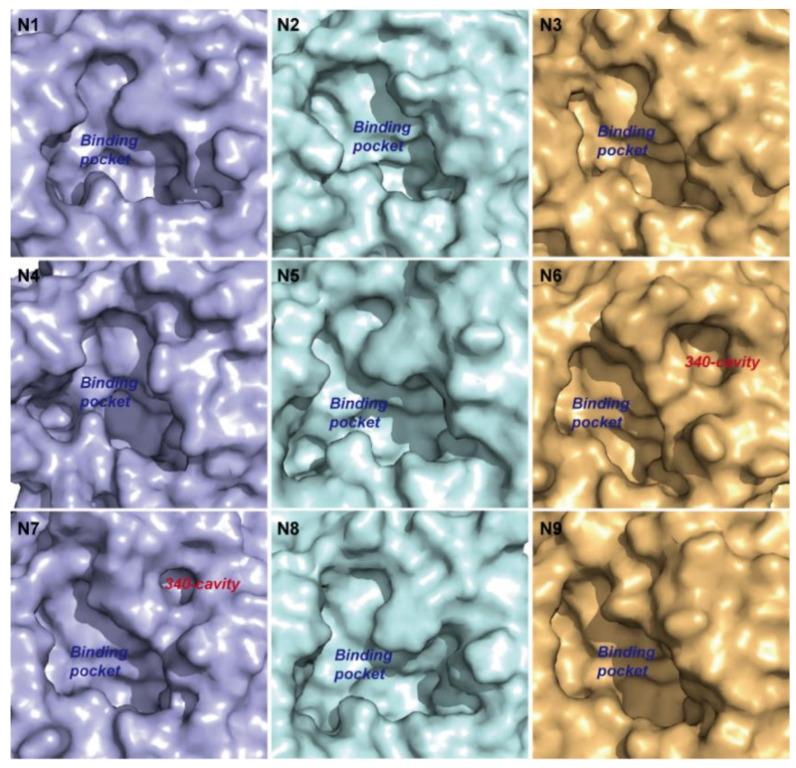
Surface of the NA binding pocket in different subtypes. Surface of the binding pocket in the crystal structures of different NA subtypes. N6 and N7 have an open 340-cavity in the crystal structures.

Type-1 NAs comprise N1, N3, N4, N5, and N8. Although no 340-cavity was observed in the crystal structures of type-1 NAs (Figure 2), their 340-loops transitioned to open conformations during simulation. In particular, one local minimum indicating the open conformation of the 340-cavity in N1 and N4 was discovered, and the local minimum is centered around 75 and 100 Å^3^ in N1 and N4, respectively (Figure 1A). For N3, N5, and N8, local minima indicating a larger 340-cavity with volumes 232, 154, and 175 Å^3^ are shown in Figure 1B. Type-2 NAs comprise N2 and N9, wherein almost no 340-cavity forms during simulation (Figure 1C). Type-3 NAs comprise N6 and N7, of which the crystal structures already exhibit an open 340-cavity (Figure 2). Based on the PMF maps, local minima with volumes of 107 and 154 Å^3^ are associated with the lowest free energy in N6 and N7, respectively (Figure 1D). PMF maps illustrate that the 340-cavity of N1, N3, N4, N5, N6, N7, and N8 are able to exhibit the open configuration, while N2 and N9 prefer the closed conformation of the 340-cavity.

### 2.2. 340-Cavity Formation Is Governed by the Side-Chain Orientation of Residue 344

To identify the conformational alterations that induce 340-cavity opening, root mean squared fluctuation (RMSF) calculation and clustering analysis of each NA subtype were carried out. The 340-loop residues of each NA subtype are listed in Table 1. RMSF values indicate that residues 342, 343, and 344 were associated with larger flexibility than that of residue 345, 346, and 347 in the 340-loop of type-1 NAs (N1, N3, N4, N5, and N8), while RMSF values of the 340-loop residues in type-2 NAs (N2 and N9) showed little discrepancies (Table 2). The RMSF results suggest that the open conformation of the 340-cavity in type-1 NAs may be controlled by residues 342–344 in the 340-loop.

**Figure 3:**
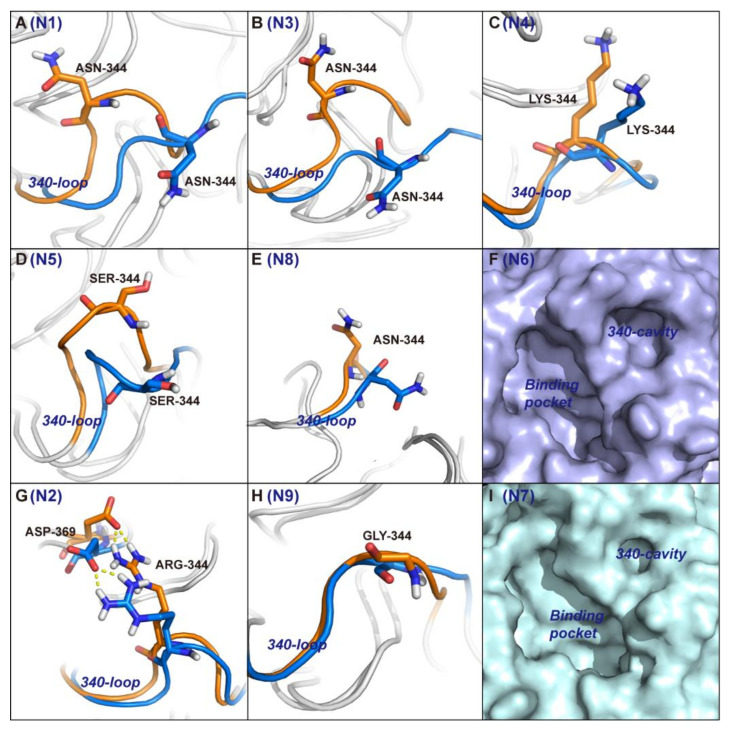
Mechanism for 340-cavity formation. Cluster analysis based on the 340-loop root mean squared fluctuation was performed. Cluster centers representing the open conformation of the 340-cavity were superimposed on the crystal structures of type-1 and type-2 NAs. Panels (**A**–**E**) show a structural comparison between the cluster center and crystal structure of the 340-loop in type-1 NAs N1, N3, N4, N5, and N8. Panels (**G**,**H**) show a structural comparison between the cluster center and crystal structure of the 340-loop in type-2 NAs N2 and N9. The 340-loops of cluster centers and crystal structures are colored in blue and orange, respectively. Panels (**F**,**I**) illustrate the 340-cavity and binding pocket of type-3 NA N6 and N7 with surface representation.

The 340-loop of the representative structures in open configuration displays a great deviation from its locations in the crystal structures for type-1 NAs. Moreover, the side-chain of residue 344 was discovered to be oriented to the opposite direction, which left a space for the formation of 340-cavity and induced the transition state of the 340-loop from the closed to the open conformation in type-1 NAs (Figure 3A–E).

In contrast, ARG-344 in type-2 NA N2 never changed orientation from its initial position. The strong salt bridge interaction between ARG-344 and ASP-369 in N2 locked the side-chain of ARG-344 and intensified the rigidity of the 340-loop, leading to an always-closed conformation of the 340-loop (Figure 3G). In N9, the 344th position was occupied by the amino acid glycine, which has no side-chain, and the 340-loop maintained its starting crystal structure conformation during the entire simulation (Figure 3H). In N6 and N7, the comparison between the crystal structure and representative structure is not displayed because the 340-cavity in the crystal structures already exhibit an open conformation (Figure 3F,I).

### 2.3. The 340-Loop Is Involved in the SA Binding Pathway

**Figure 4:**
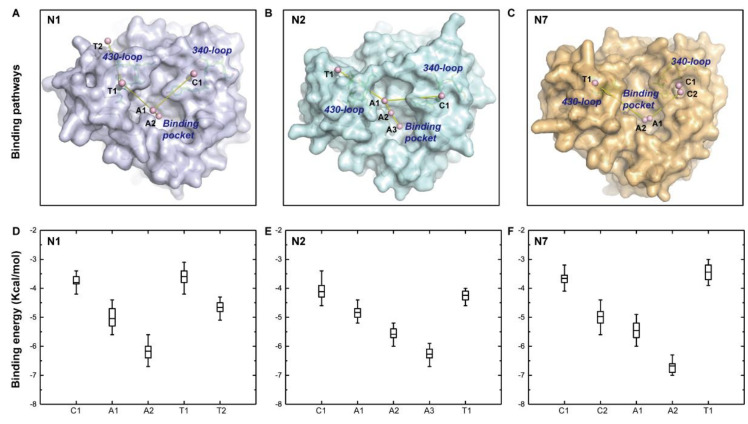
Binding pathway of sialic acid (SA) and distribution of binding energies along the pathway. The two lowest binding energy pathways of sialic acid (SA), the *climbing path* and *tunneling path*, are illustrated in N1 (**A**), N2 (**B**), and N7 (**C**). The harbors of the climbing path, active site, and tunneling path were abbreviated as C, A, and T, respectively. Panels (**D**–**F**) represent binding energies in accordance with the harbor sites in N1, N2, and N7, respectively. Boxes represent binding energies between 25% and 75% of the cluster components, lines within the boxes represent mean values, and vertical bars represent binding energies within 1.5 interquartile range (IQR).

The discovery of the 340-cavity in seven of nine NA subtypes via simulation indicates that small molecule compounds that target this cavity could have pan-NA activity. The resultant compounds would interfere with NA function if the 340-loop or 340-cavity is involved in natural SA substrate binding. To figure out the function of the 340-loop in SA binding, sliding binding-box docking was performed on N1, N2, and N7, representing the three types of NAs. The docking procedure can be observed in Figure S1. Two lowest binding energy pathways of SA were proposed in the three NA subtypes, designated the *climbing path* and the *tunneling path*. This method was inspired by a previous sliding binding-box docking study which revealed the binding mechanism of a NA inhibitor [13]. The binding path of SA and the averaged binding energy of cluster components are illustrated in Figure 4.

In the climbing path, the natural substrate SA did a brief layover in the 340-loop region in the three NAs. Although the 340-loop in the N1 and N2 crystal structures displayed a closed conformation, SA was still held in the 340-loop region for a time while approaching the final binding pocket in both NAs (Figure 4A,B). There were two harbors in the climbing path of SA in N7 since the 340-loop exhibited an open conformation in the N7 crystal structure (Figure 4C). After a brief layover at the 340-loop region, the SA continued to move to the active site via two (A1 and A2 in N1 and N7) or three steps (A1, A2, and A3 in N2). The large decrease in binding energy made this movement energetically favorable (Figure 4D–F).

In the tunneling path, the 430-loop (residues 430–433) adjacent to the binding pocket was discovered to act as a harbor that facilitated SA landing in N1, N2, and N7. The average binding energies of the cluster centers in the tunneling path were larger than that of cluster centers in the climbing path (Figure 4D–F). Thus, the probability of SA binding via the tunneling path was lower than that of the climbing path, a finding in good agreement with a previous study [13]. The SA binding mechanisms determined here suggest that the 340-loop is an important motif in shepherding SA to the final binding pocket, and this finding indicates that the 340-cavity is a potential target pocket for drug discovery.

### 2.4. A Calcium Ion Controls the Stability of the 340-Loop

**Figure 5:**
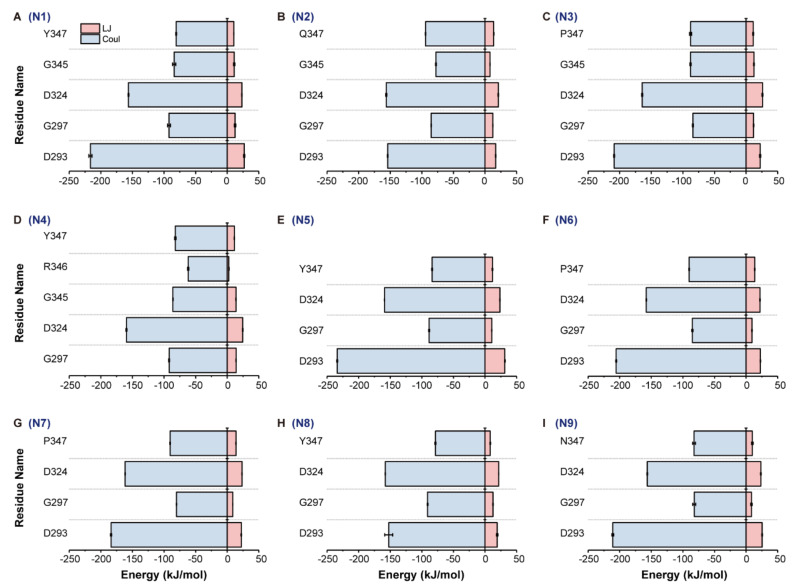
Interaction energy between the conserved calcium ion and interacted residues in all NA subtypes. Interaction energy, Lennard-Jones (LJ) and Coulomb (Coul), between the calcium ion and interacted residues was calculated based on the whole trajectory of each NA subtype. Panels (**A**) to (**I**) represent the LJ and Coul interactions of N1 to N9, respectively.

The 340-loop was found to be involved in the SA binding mechanism. Therefore, the conformation and stability of the 340-loop need to be carefully characterized. A conserved calcium ion found to influence the activity and thermostability of NA is located near the 340-loop [14,15]. At least one residue on the 340-loop interacts with this cation ion (Table 3). The interaction energy between the calcium ion and residues in Table 3 contains two parts, the Lennard-Jones (LJ) and Coulomb (Coul) interactions. The LJ and Coul interactions between the calcium ion and interacted residues of the nine NA subtypes were well-preserved during simulations, since error values of block averages are relatively small (Figure 5).

**Figure 6:**
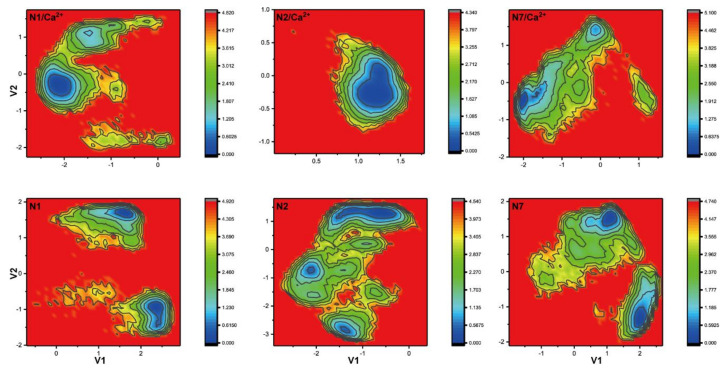
Free energy landscape of the 340-loop from dihedral principle component analysis. Free energy landscapes of the 340-loop in N1, N2, and N7 calcium-bound and calcium-free systems were obtained using dihedral principle component analysis (dPCA).

To further characterize the conformational changes of the 340-loop due to influence of the calcium ion, dihedral principal components analysis (dPCA) of the 340-loop in the calcium-bound and calcium-free systems was performed. Free energy landscapes of N1, N2, and N7, representing the three 340-cavity types of NA, are shown in Figure 6. It is evident that the free energy landscapes of the 340-loop in the calcium-bound and calcium-free systems of all NA subtypes are distinct from each other, indicating disparate configurations of the 340-loop (Figure S2). Specifically, only one local minimum was observed in the calcium-bound NA systems, while two or more local minima were observed in the calcium-free systems. Interestingly, comparing free energy landscapes of the 340-loop in the calcium-bound systems, configurations of the 340-loops in N1 and N7 experienced a wider space than that in N2 system, which is directly related to the 340-cavity volume calculation. In the volume calculation, the N2 340-loop maintained its closed conformation during the whole trajectory, while the N1 340-loop transitioned from closed to open state, and the N7 340-cavity opened to a larger extent during the simulations. The dPCA analysis revealed that the conserved calcium ion plays an important role in maintaining the stability of the 340-loop, and removal or disruption of this cation ion could severely influence the 340-loop conformation.

## 3. Discussion

The 340-loop was discovered to be a canonical accommodating pocket along SA’s binding mechanism to NA across multiple NA subtypes. This implicates that the SA binding process may be influenced or terminated if the 340-loop is reconfigured or blocked from its conventional configuration. Our results are consistent with a previous study in which the NA inhibitor binding pathway was explored and oseltamivir was found to loosely localize between the two loops 294-NWH-296 and 345-GAY-347 [13]. Although the authors stated that this finding may not be prevalent in other NAs, we have found that the 340-loop participates in SA binding of N1, N2, and N7, which represent three types of NAs. The 340-cavity was found in seven of nine NA subtypes, which indicated that this cavity could guide the design of robust novel ligands which function as NA inhibitors by interfering with the SA binding mechanism. Our study provides more evidence that supports the 340-cavity as a potential drug target.

Additionally, the conserved calcium ion was found to maintain the stability of the 340-loop due to its intimate contact with residues on the loop. Specifically, the backbone carbonyl group of residue Y/P/Q/N347 stably interacts with the calcium ion in all NA subtypes (Table 3 and Figure 5). Y347 in N1 was identified as the key framework residue that can “clamp” the ligand into a favorable binding pose in one study [16]. Two other studies found that residue 347 could mediate oseltamivir on the NA surface via a hydrogen bond [13,17]. All these findings, including our own, indicate that the conserved calcium ion plays an important role in stabilizing the conformation of the 340-loop, thus facilitating SA/drug binding. Therefore, disruption of the calcium ion binding can also influence NA function and thus stop viral release.

Moreover, the 430-loop was discovered to be a common stop in the SA tunneling pathway of the three NA subtypes. The displacement of the 430-loop was found to induce a larger opening of the binding pocket based on crystal structural analysis and MD simulations [18,19]. The 430-loop was also predicted to be a novel druggable hot spot by a computational solvent mapping study [20]. Our study provides new evidence that supports the 430-loop as a potential drug-targeting region.

In conclusion, this study demonstrates the plasticity of the 340-loop in different NA subtypes and the importance of the conserved calcium ion in influencing the stability of the 340-loop. The open 340-cavity discovered in the seven NA subtypes and the mechanism of 340-cavity formation offer new structural insights for antiviral drug development. Moreover, the 340-loop’s role as a canonical stop in the SA climbing path suggests it can function as a novel drug-binding hotspot for combating influenza viruses.

## 4. Methods 

### 4.1. MD Simulation Details

Normal MD simulations of nine subtypes of NA were performed, and the X-ray crystal structures of N1 to N9 were taken from PDB 2HTY, 1NN2, 4HZV, 3HTV, 3SAL, 4QN4, 4QN3, 2HT5, and 4MWJ, respectively [10,11,21,22,23,24]. All the NA structures were crystallized with the conserved calcium ion, and the calcium ion coordinated with interacted residues (Table 3) was preserved in the calcium-bound system and deleted in the calcium-free system. The simulation time for each NA subtype with or without calcium ion was 100 ns, with total simulation of 1.8 μs in this study. All the systems were solvated with TIP3P waters in an octahedral box with periodic boundary conditions [25]. Sodium and chloride ions were added to a concentration of 100 mM. The GROMACS and Amber99SB force field were used in all simulations [26]. All systems were first equilibrated for 5 ns by restraining all the heavy atoms, then normal MD simulations were performed in an isothermal, isobaric ensemble (300 K, 1 bar) for 100 ns. Bond length constraints were applied to all bonds that contained hydrogen atoms based on the LINCS protocol [27]. An integration step of 0.002 ps was used in all the simulations. Electrostatic interactions were treated with Particle Mesh Ewald with a cutoff of 0.9 nm with grid spacing for the FFT grid < 0.12 nm [28].

### 4.2. Sliding Binding-Box Docking

Sliding binding-box docking was performed using Autodock Vina software [13,29]. The N1, N2, and N7 crystal structures were treated as receptors, and the direction from the binding pocket to the exposed solvent was defined as the z-axis. Thirteen overlapping grid boxes along the z-direction were generated, wherein the z plane covers the binding pocket and the xy planes cover the entire protein area. Each grid box was set to size 48 × 48 × 10.5 Å, with an overlap of 2 Å in the z-direction. In detail, the x and y coordinates of the overlapped grid boxes were kept the same as those of the thirteen grid boxes in the Autodock setup procedure, while the z coordinate was increased by 2 Å for each grid box. The representative image of sliding binding-box docking is shown in Figure S1. Sialic acid (SA) was prepared as the ligand. An exhaustiveness value of 20 was set in Autodock Vina. The possible binding mechanism of SA was revealed by k-means clustering: the x, y, and z coordinates of the center of mass of the ligand and the docking score were treated as coordinates in four dimensions, and the docking score was weighted by a factor of 0.7. The number of cluster centers was set at 5.

### 4.3. 340-Volume Calculation

The volume of the 340-cavity was calculated using POVME [30]. NA structure snapshots were extracted from the trajectory of each NA subtype with the calcium ion every 5 ps and superimposed onto the reference structure (N7) with a pregenerated 3D-grid representing the 340-cavity. The volume was calculated by counting the grid points located in the 340-cavity.

### 4.4. Dihedral Principal Components Analysis (dPCA)

Before dPCA, all backbone dihedral angles of the 340-loop in N1 to N9 were collected from trajectories every 1 ps [31]. dPCA was performed on the angles collected from each NA in the calcium-bound and calcium-free systems. All calculated snapshots were projected to the first two eigenvectors to get the first two principal components to make a two-dimensional free energy landscape.

## Figures and Tables

**Table 1:** Residues of the 340-loop in each NA subtype.

NA Subtype	340-Loop Residues
N1	342-SSNGAY-347
N2	342-NERGTQ-347
N3	342-NVNGGP-347
N4	342-NGKGRY-347
N5	342-GGSGTY-347
N6	342-GGSPDP-347
N7	342-GSPGAP-347
N8	342-G-NQGY-347
N9	342-YPGNNN-347

N8 (PDB 2HT5) lacks residue 343.

**Table 2:** Root mean squared fluctuation (RMSF) for 340-loop residues over the whole simulation in type-1 and type-2 NAs.

Residue No.	RMSF of Type-1 NAs (nm)					Type-2 NAs (nm)	
	N1	N3	N4	N5	N8	N2	N9
342	0.2524	0.3295	0.1536	0.1119	0.1072	0.1234	0.1072
343	0.1571	0.3867	0.2273	0.1326	N.A.	0.0775	0.1057
344	0.2590	0.3599	0.1575	0.2121	0.2154	0.0969	0.1396
345	0.0973	0.0982	0.0759	0.1113	0.1384	0.0612	0.1474
346	0.0742	0.0905	0.0973	0.0902	0.0839	0.0598	0.1623
347	0.1240	0.0861	0.1321	0.1659	0.1365	0.0709	0.1496

**Table 3:** Residues interact with the conserved calcium ion in each NA subtype.

NA Subtype	Residues Interact with the Calcium Ion
N1	D293, G297, D324, **G345**, **Y347**
N2	D293, G297, D324, **G345**, **Q347**
N3	D293, G297, D324, **G345**, **P347**
N4	G297, D324, **G345**, **R346**, **Y347**
N5	D293, G297, D324, **Y347**
N6	D293, G297, D324, **P347**
N7	D293, G297, D324, **P347**
N8	D293, G297, D324, **Y347**
N9	D293, G297, D324, **N347**

Residues in bold font are 340-loop residues.

## Supplementary Materials

Supplementary Materials can be found at https://www.mdpi.com/1422-0067/21/16/5655/s1.

## Abbreviations

dPCA	dihedral principle component analysis
MD	Molecular dynamics
NA	Neuraminidase
PMFRMSF	Potential of mean forceRoot mean squared fluctuation
