# Supplementary material for: Plasticity of the 340-Loop in Influenza Neuraminidase Offers New Insight for Antiviral Drug Development
Source: Int J Mol Sci. 2020 Aug 6;21(16):5655. doi: 10.3390/ijms21165655 (PMC7460844; doi:10.3390/ijms21165655)
Supplement: Supplementary file 1 [file ijms-21-05655-s001.zip › ijms-885545-supplementary.pdf]

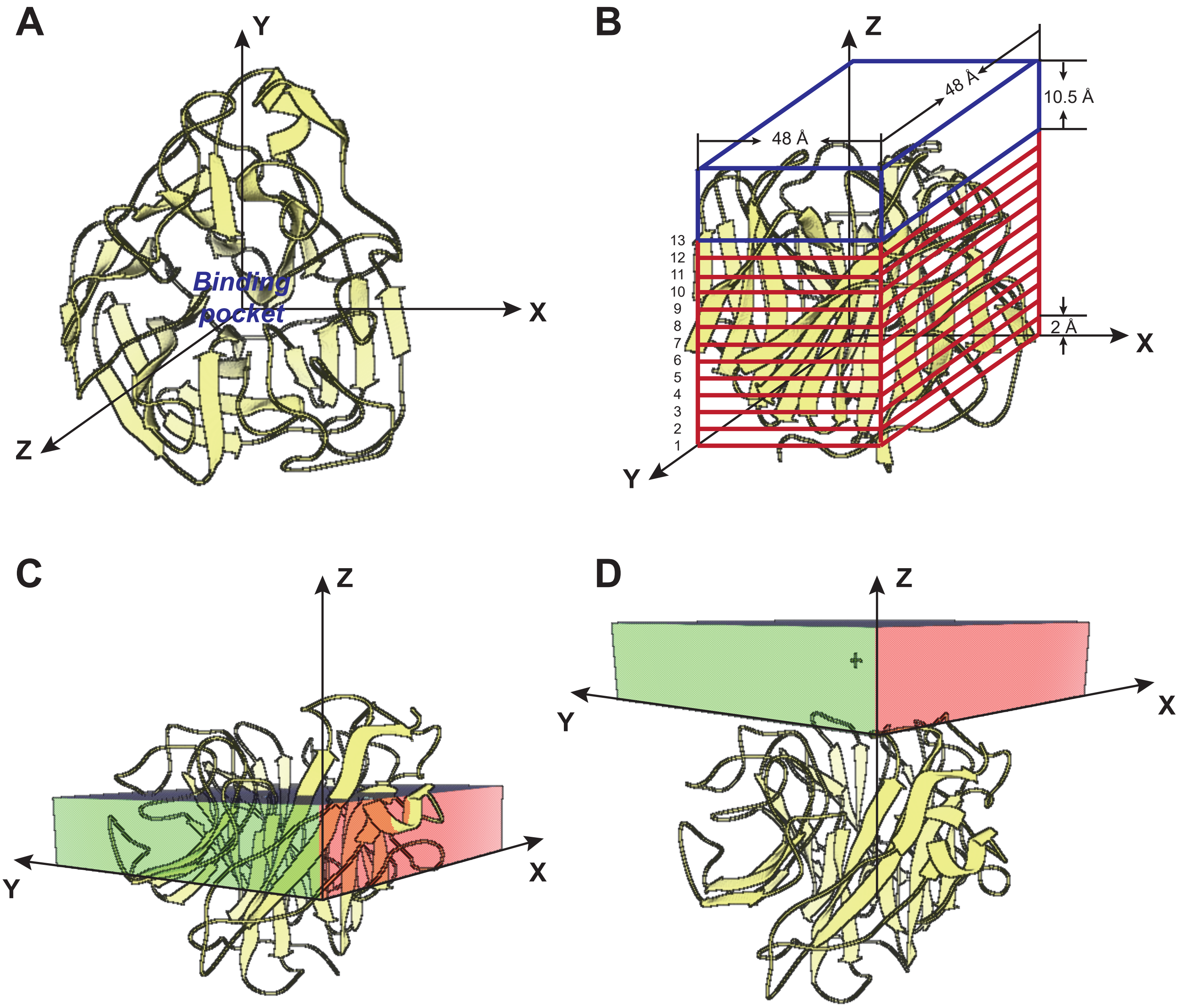

Figure S1. Representative Scheme of sliding binding-box docking. NA structure (N1) is shown in cartoon. Panel (A) displays the binding pocket of NA and the x, y, and z axis in the sliding binding-box docking process. Panel (B) provides the scheme of the grid box setup process. Box with red lines indicate overlapped parts in z-direction, box with blue lines represent the top box numbered with 13. Panel (C) and (D) represent the bottom (number 1) and top (number 13) grid boxes in the real sliding-binding box procedure, respectively.

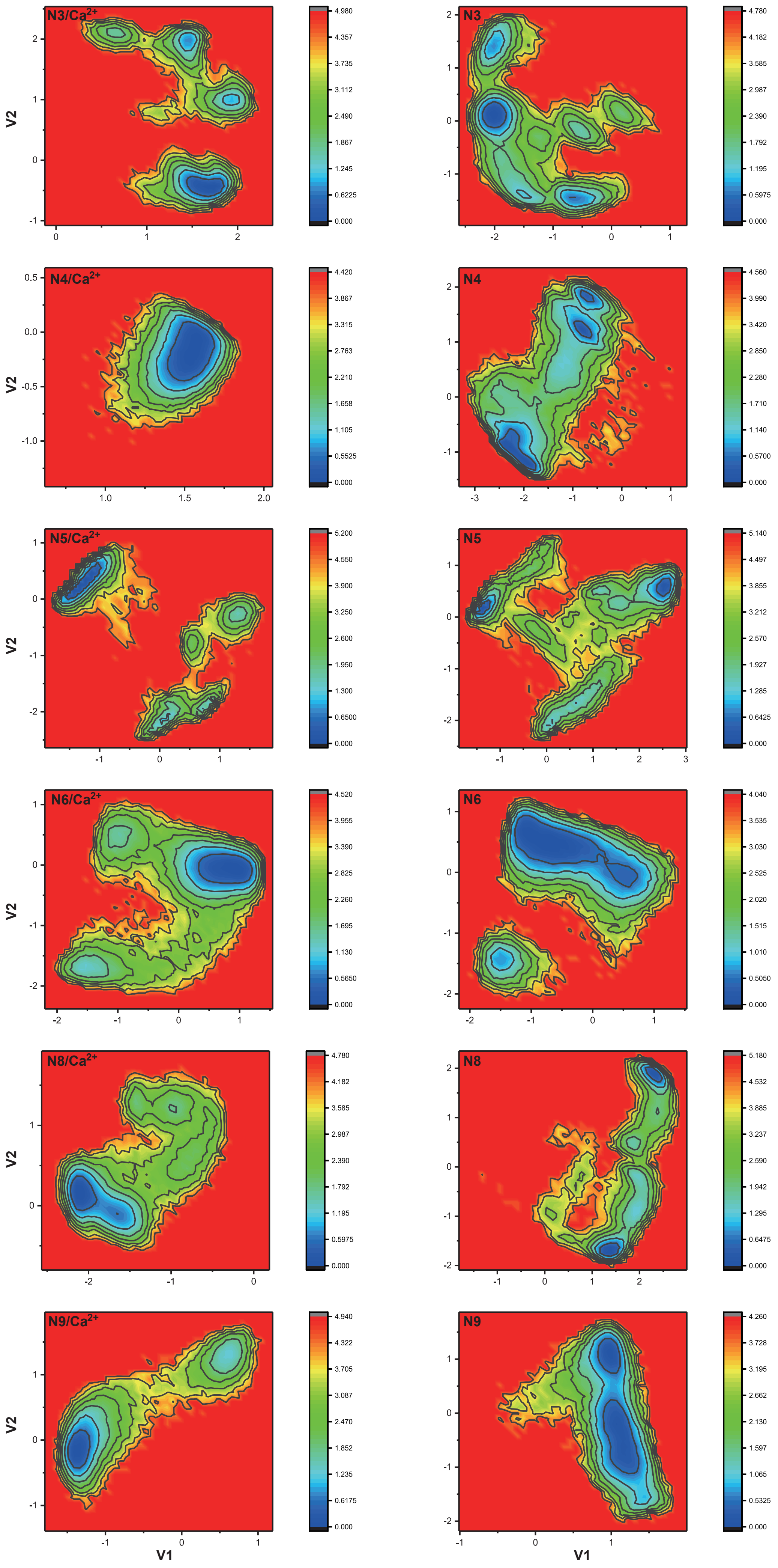

Figure S2. Free energy landscapes of the 340-loop in N3, N4, N5, N6, N8, and N9 calcium-bound and calcium-free systems were obtained using dihedral principle component analysis (dPCA).
